# 2D perfusion DSA with an open-source, semi-automated, color-coded software for the quantification of foot perfusion following infrapopliteal angioplasty: a feasibility study

**DOI:** 10.1186/s41747-020-00176-z

**Published:** 2020-09-02

**Authors:** George C. Kagadis, Stavros Tsantis, Ilias Gatos, Stavros Spiliopoulos, Konstantinos Katsanos, Dimitris Karnabatidis

**Affiliations:** 1 Department of Medical Physics, School of Medicine, University of Patras, GR 26504 Rion, Greece; 2 Department of Imaging Physics, The University of Texas MD Anderson Cancer Center, Houston, TX 77030 USA; 3 2nd Department of Radiology, School of Medicine, “ATTIKON” University Hospital, National and Kapodistrian University of Athens, GR 12461 Athens, Greece; 4 Department of Radiology, School of Medicine, University of Patras, GR 26504 Rion, Greece

**Keywords:** Angiography (digital subtraction), Endovascular procedures, Foot, Perfusion, Peripheral arterial disease

## Abstract

**Background:**

Foot perfusion has been recently implemented as a new tool for optimizing outcomes of peripheral endovascular procedures. A custom-made, two-dimensional perfusion digital subtraction angiography (PDSA) algorithm has been implemented to quantify outcomes of endovascular treatment of critical limb ischemia (CLI), assist intra-procedural decision-making, and enhance clinical outcomes.

**Methods:**

The study was approved by the Hospital’s Ethics Committee. This prospective, single-center study included seven consecutive patients scheduled to undergo infrapopliteal endovascular treatment of CLI. Perfusion blood volume (PBV), mean transit time (MTT), and perfusion blood flow (PBF) maps were extracted by analyzing time-intensity curves and signal intensity on the perfused vessel mask. Mean values calculated from user-specified regions of interest (ROIs) on perfusion maps were employed to evaluate pre- and post-endovascular treatment condition. Measurements were performed immediately after final PDSA.

**Results:**

In total, five patients (aged 54 ± 16 years, mean ± standard deviation) were analyzed, as two patients were excluded due to significant motion artifacts. Post-procedural MTT presented a mean decrease of 19.1% for all patients and increased only in 1 of 5 patients, demonstrating in 4/5 patients an increase in tissue perfusion after revascularization. Overall mean PBF and PBV values were also analogously increased following revascularization (446% and 69.5% mean, respectively) and in the majority of selected ROIs (13/15 and 12/15 ROIs, respectively).

**Conclusions:**

Quantification of infrapopliteal angioplasty outcomes using this newly proposed, custom-made, intra-procedural PDSA algorithm was performed using PBV, MTT, and PBF maps. Further studies are required to determine its role in peripheral endovascular procedures (ClinicalTrials.gov Identifier: NCT04356092).

## Key points

- A method for perfusion analysis of two-dimensional digital subtraction angiography (DSA) images was developed.
- Mean transit time, blood volume, and blood flow were quantified on subtraction images.
- All two-dimensional perfusion metrics were overall improved after infrapopliteal angioplasty.
- DSA perfusion analysis holds promise for quantification of angioplasty results in terms of tissue perfusion analysis.

## Background

Peripheral arterial disease (PAD) affects over 200 million persons worldwide and is considered a major cause of increased hospitalization, limb amputation, and mortality risk [1]. The weighted mean age-standardized prevalence and incidence of outpatient PAD in the USA have been recently estimated to be 11.8% and 22.4/1,000 person-years, respectively [2]. Minimal invasive endovascular treatment has become a mainstay in the treatment of PAD, due to high clinical success and low complication rates [3, 4]. Especially for the management of critical limb ischemia (CLI), the most advanced stage of PAD which usually affects a more aged population with severe comorbidities and high surgical risk, endovascular treatment constitutes the best treatment option to avoid major limb amputation in significant percentages of everyday clinical practice [5, 6]. Approximately 5–10% of PAD patients will progress to CLI [6], while the incidence of CLI in the Western population is estimated to be approximately 500 to 1,000 per million per year [7] and is anticipated to increase in the years to come, due to the concomitant increase of risk factors associated with the disease such as diabetes, metabolic syndrome, and advanced age [7–10].

Despite the recent vast development of various endovascular devices and techniques, several issues crucial for the improvements of clinical outcomes remain unresolved such as the quantification of limb perfusion following endovascular treatment, which is the primary goal of revascularization [11]. An objective and quantifiable, tissue perfusion imaging approach could assist intra-procedural decision-making on whether perfusion has been adequately improved or revascularization of further arteries/lesions is required, and provide a significant measurable metric for the documentation of endovascular treatment outcomes that could be correlated with limb prognosis. Recent research on this field has produced encouraging results, while commercially available products have also been developed [12, 13].

A custom-made, two-dimensional perfusion digital subtraction angiography (DSA) algorithm has been designed and implemented towards foot perfusion quantification following endovascular treatment of CLI in order to quantify endovascular treatment outcomes, assist intra-procedural decision-making, and enhance clinical outcomes.

## Methods

At first, a three-dimensional (3D) segmentation method based on the fuzzy C-means clustering algorithm [14] was employed to extract the vessels that have been perfused, and produce a perfused vessel mask both on pre- and post-operation DSA image sequences. Then, perfusion blood volume (PBV), mean transit time (MTT), and perfusion blood flow (PBF) maps were extracted by analyzing time-intensity curves (TICs) and signal intensity on the perfused vessel mask. Finally, mean values calculated from user-specified regions of interest (ROIs) on perfusion maps are employed to evaluate patient’s pre- and post-endovascular treatment condition.

The study was approved by the Hospital’s Ethics Committee. This was a prospective, single-center study investigating 2D perfusion DSA using newly developed, non-commercially available, semi-automated, color-coded software for the quantification of foot perfusion following infrapopliteal angioplasty for the treatment of CLI (ClinicalTrials.gov Identifier: NCT04356092). All procedures were performed using the Philips Allura Xper FD20 x-ray angiography system (Philips Healthcare, Best, The Netherlands).

Inclusion criteria were as follows: (i) patients scheduled to undergo infrapopliteal angioplasty or stenting, or both, as part of their standard treatment for Rutherford-Becker class 4 to 6 CLI; (ii) written consent to participate to the specific clinical protocol. There were no restrictions in lesion type (occlusion or stenosis), lesion length, or anatomical location (below or above the ankle). Exclusion criteria were as follows: (i) acute limb ischemia, (ii) contraindication to dual antiplatelet therapy using clopidogrel 75 mg and acetylsalicylic acid 75–100 mg once daily for 6 months, and (iii) impaired mental capacity. In total, seven consecutive patients fulfilled the abovementioned criteria and were included in the study [5]. DSA image acquisition protocol was as follows: the target limb was immobilized to the angiographic table using a soft strap as described elsewhere [13]. All procedures were performed using local anesthesia (lidocaine 2% up to 20 mL). In patients experiencing pain (5/7 cases) when lying on the table, conscious sedation using fentanyl (50–200 μg) and midazolam (up to 5 mg) was used.

An antegrade common femoral artery access was used in all patients followed by the deployment of 5- or 6-Fr arterial sheaths. A semi-lateral foot projection was preferred, and the pre-revascularization DSA of the foot was performed via a 5-Fr angiographic catheter (Vertebral, Radifocus® Glidecath®, Terumo Europe, Belgium) placed at the distal third of the popliteal artery and subsequent injection of 10 mL of non-ionic contrast medium (Visipaque 320, General Electric Health care A.S., Oslo, Norway) using a power injector with a flow rate of 5 mL/s, as per standard departmental protocol. Revascularization was performed using standard angiographic catheters mainly the above described 5-Fr vertebral catheter, 0.014’ or 0.018’ guidewires and low-profile dedicated infrapopliteal balloons measuring between 2 and 3.5 mm in diameter and 40 to 220 mm in length. Intraluminal lesion crossing was always initially attempted, but the subintimal technique was also implemented if required. Stenting would only be performed as a bail out following suboptimal angioplasty. Following revascularization of one or more infrapopliteal arteries, the catheter was placed at the same popliteal segment and post-procedural DSA of the foot was performed following the exact pre-revascularization injection protocol at the same semi-lateral projection. Procedural success was defined as the identification of at least one of the targeted infrapopliteal arteries patent to the distal foot with less than 30% remaining stenosis according to visual estimation at final DSA [7]. Technical success was defined as successful image post-processing resulting in quantification of infrapopliteal angioplasty. Patients’ demographical data and procedural details were recorded and are presented in Table 1. The 2D perfusion imaging and analysis of the Digital Imaging and COmmmunications in Medicine (DICOM) files were performed after revascularization, *i.e.,* immediately after the final DSA. Perfusion analysis was performed by one of the software developers (S.T.).

**Table 1:** Patients’ demographic and outcomes

Patient	Age (years)	Gender	Risk factors	Wound description	Baseline lesion	Arteries treated	Technical success	Pedal arch status	6-month limb salvage
1	45	Male	Type II diabetes	Rest pain	ATA occlusion	ATA	Yes	Incomplete	Yes
2	55	Female	Type II diabetes hyperlipidemia hypertension	Big toe wet gangrene	PA occlusion. Pedal arch occlusion	PA and pedal arch balloon angioplasty	Yes	Complete	Yes
3	72	Male	Type II diabetes hyperlipidemia hypertension	Heel wet gangrene and rest pain	TB/PA and PTA occlusion	TB/PA and PTA balloon angioplasty	Yes	Complete	Yes
4	54	Female	Diabetes, hyperlipidemia	5th toe gangrene	ATA/TB/PA stenosis	ATA/TB/PA stenosis balloon angioplasty	Yes	Complete	Yes
5	28	Male	Type I diabetes dialysis smoking	Transmetatarsal amputation with recurrent gangrene	ATA single-vessel runoff stenosis	ATA balloon angioplasty	Yes	No arch depicted	No

*ATA* Anterior tibial artery, *TB* Tibioperoneal trunk, *PA* Posterior tibial artery

**Fig. 1:**
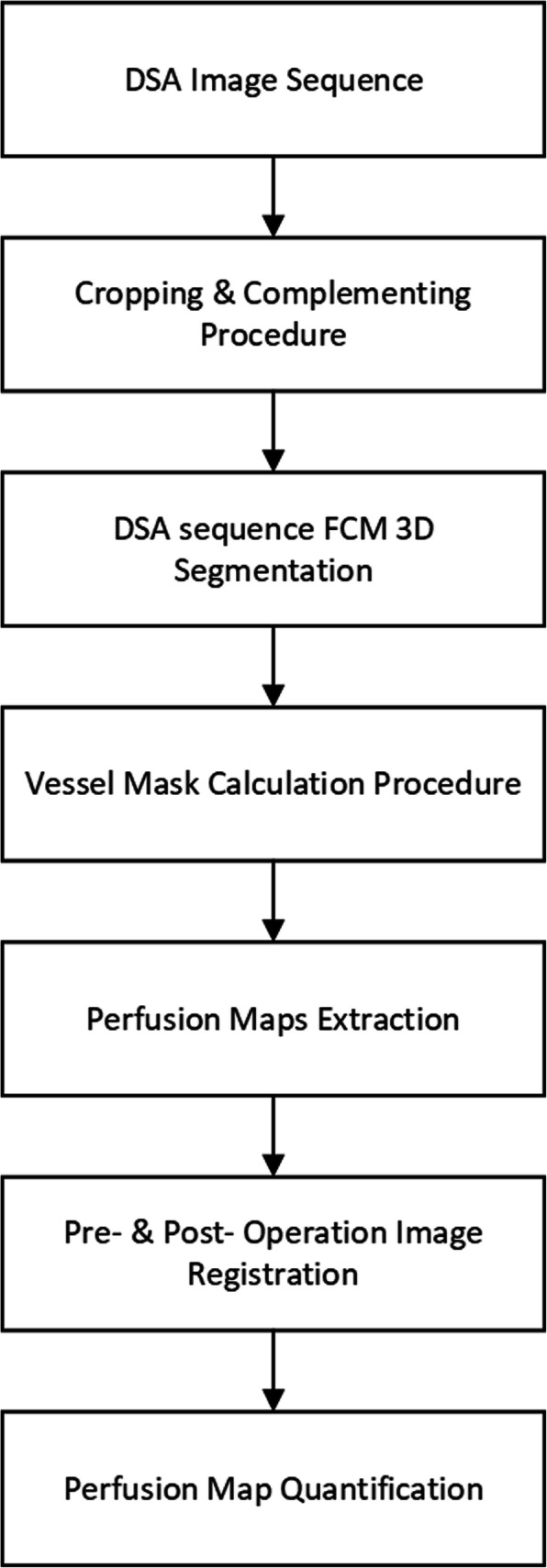
Two-dimensional perfusion digital subtraction angiography (DSA) algorithm steps. *3D* Three-dimensional, *FCM* Fuzzy C-means

**Fig. 2:**
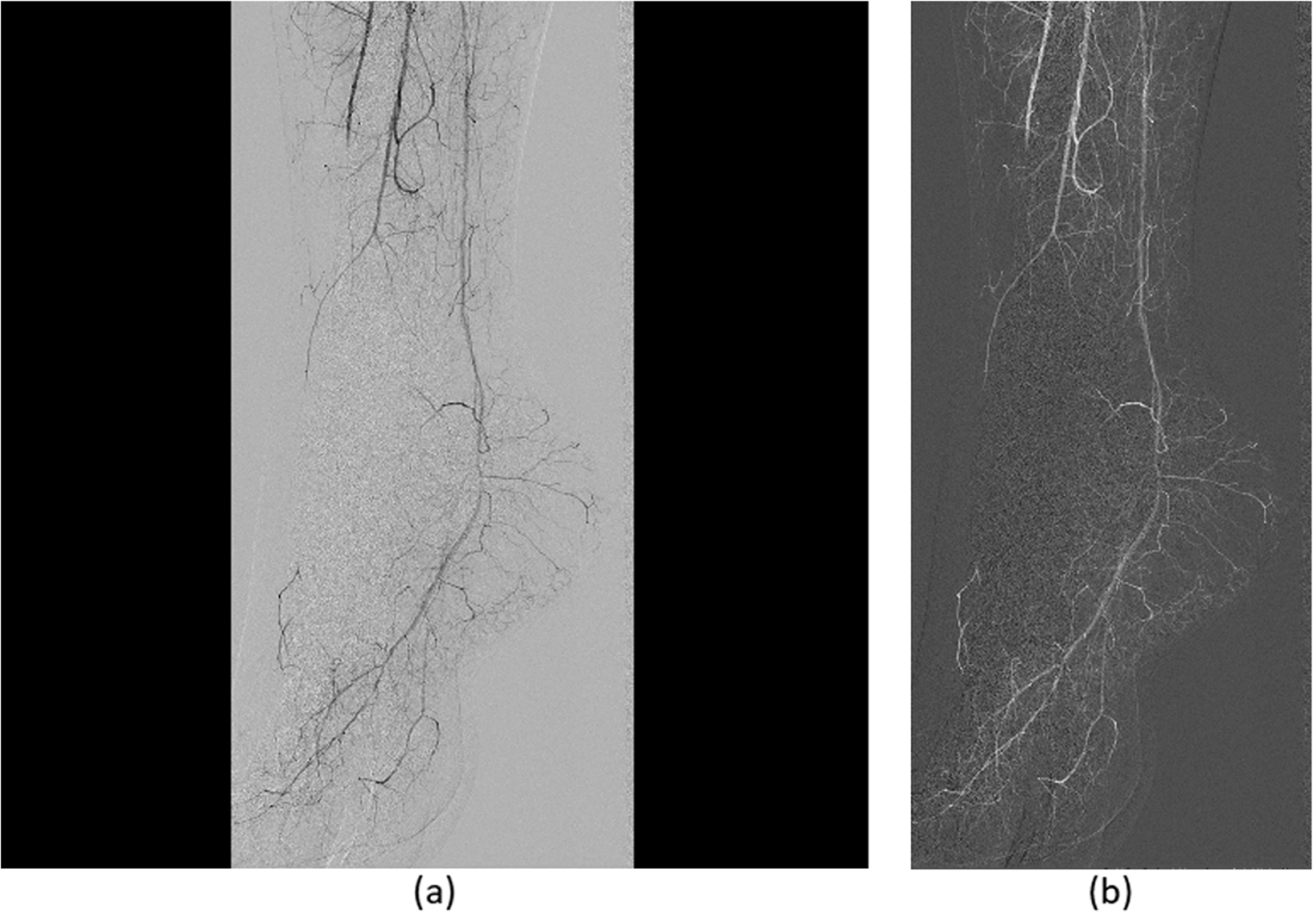
**a** Initial digital subtraction angiography image at slice 8/24 (4th second). **b** Initial image after automatic cropping and complementing

**Fig. 3:**
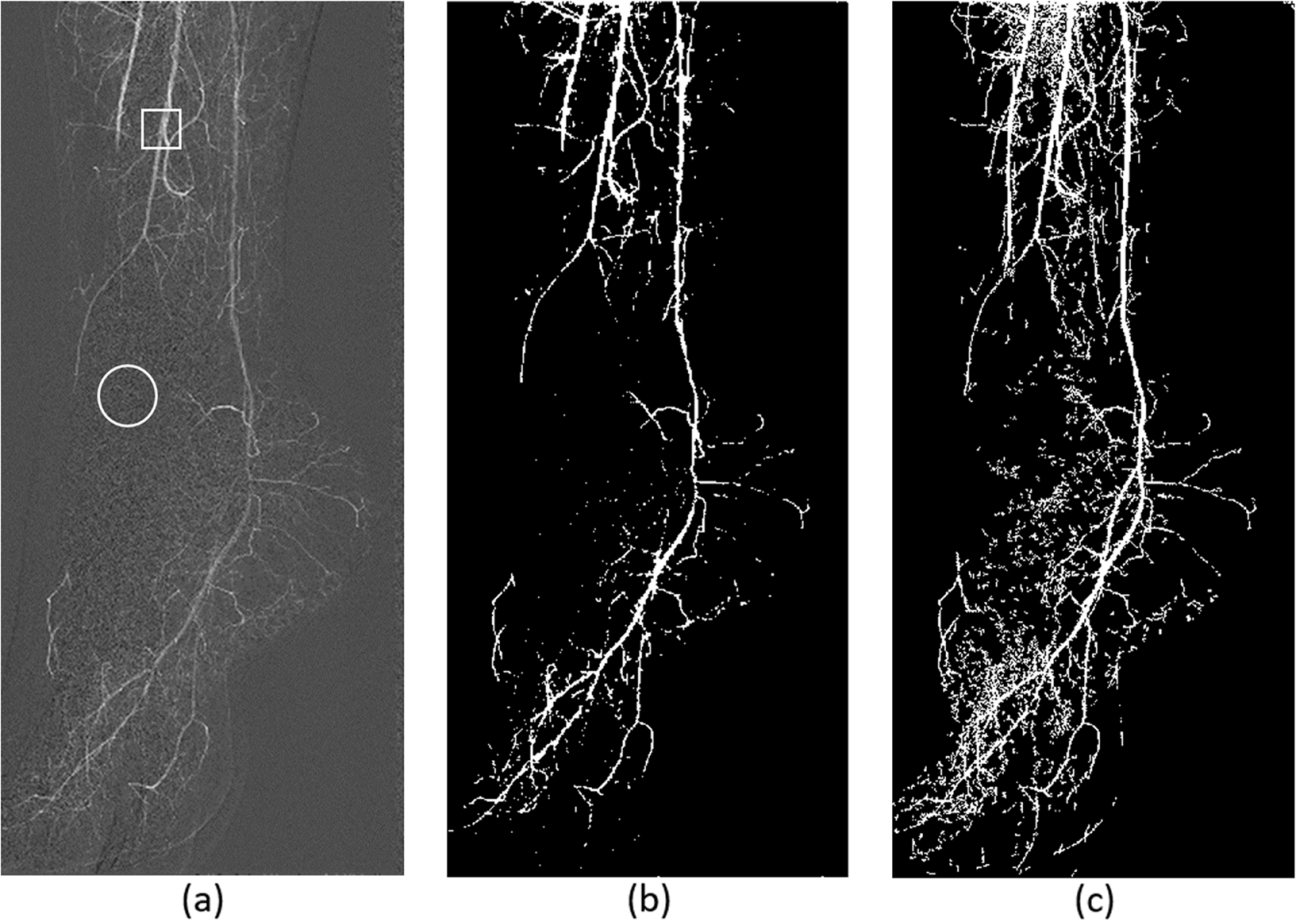
**a** User initialization regions of interest at vessel (in rectangle) and background (in circle), at a slice selected by the user 8/24 (4th second). **b** Fuzzy C-means three-dimensional segmentation result at slice 8/24 (4th second). **c** Total vessel mask after pixel voting procedure with vessel information derived from all available slices

The perfusion algorithm steps are depicted in Fig. 1. In order to extract PBV, MTT, and PBF maps, a vessel segmentation procedure was employed on all the patient examination DSA slices. This semiautomatic segmentation process at first involved a preprocessing step in which the DSA image portion with clinical information was cropped and complemented (each signal pixel value was reversed to acquire concentration values) for the subsequent processing steps (Fig. 2a). Cropping procedure involved the removal of any irrelevant borders, whereas complementing (pixel values inversion) was employed to depict perfused areas with higher values than the background (Fig. 2b). Then, an initialization procedure was utilized in which two user-defined areas (one that contained a major vessel and the second that contained the background) were fed as input into a modified 3D fuzzy C-means (FCM) clustering algorithm as described in Mandelias et al. [15] towards multislice vessel segmentation (Fig. 3a, b). This was done in order to define FCM initialization points near the two clusters’ (cluster 1: vessels; cluster 2: background) actual values and to perform more accurate and quick segmentation. ROIs’ initialization was performed on a slice specified by an interventional radiologist with 15 years of experience in endovascular procedures, providing clear view of the majority of perfused major vessels (Fig. 3a). The location and number of ROIs varied according to the angiographic features of the foot after revascularization and aimed at quantifying the reperfused areas of ischemia. The mean intensity values of the defined ROIs were used as seed points to the 3D FCM algorithm for quick and accurate perfused vessel segmentation.

The creation of an image mask that contains all perfused vessels involved a pixel voting procedure on the 3D matrix resulted from the FCM segmentation step. This process was deployed to calculate MTT, PBV, and PBF parameters only on the areas containing vessels and discard no-vessel areas to enhance the algorithm’s speed. In this voting procedure, all pixels on positions (*x*_s_, *y*_s_) that were segmented as part of a vessel at any of the slices of the segmented 3D matrix were considered as candidates for the mask to be created. An image was then created having “0” value when the pixel at position (*x*, *y*) was never labeled as “vessel” part, and “1” value when the pixel at position (*x*, *y*) was labeled at least once as “vessel” part (Fig. 3c).

To derive hemodynamic parameters from DSA images by tracer kinetic analysis, the contrast agent concentrations in various tissue and vessel compartments must be known. To derive this information, the time-intensity curves (TICs) were computed during the contrast agent bolus passage that possesses sufficient temporal resolution towards foot perfusion representation. The signal observed values during each passage were fed as input to TIC calculation. By detecting the arterial as well as the total tissue concentration as a function of time during a single transit, the PBV can be determined from the ratio of the areas under the tissue (*C*_tissue_(*t*)) and arterial (*C*_αrtery_(*t*)) concentration-time curves, respectively:

1 $$ \mathrm{CBV}=\frac{\int_0^{+\infty }{C}_{\mathrm{tissue}}(t)\mathrm{dt}}{\int_0^{+\infty }{C}_{\mathrm{artery}}(t)\mathrm{dt}} $$

**Fig. 4:**
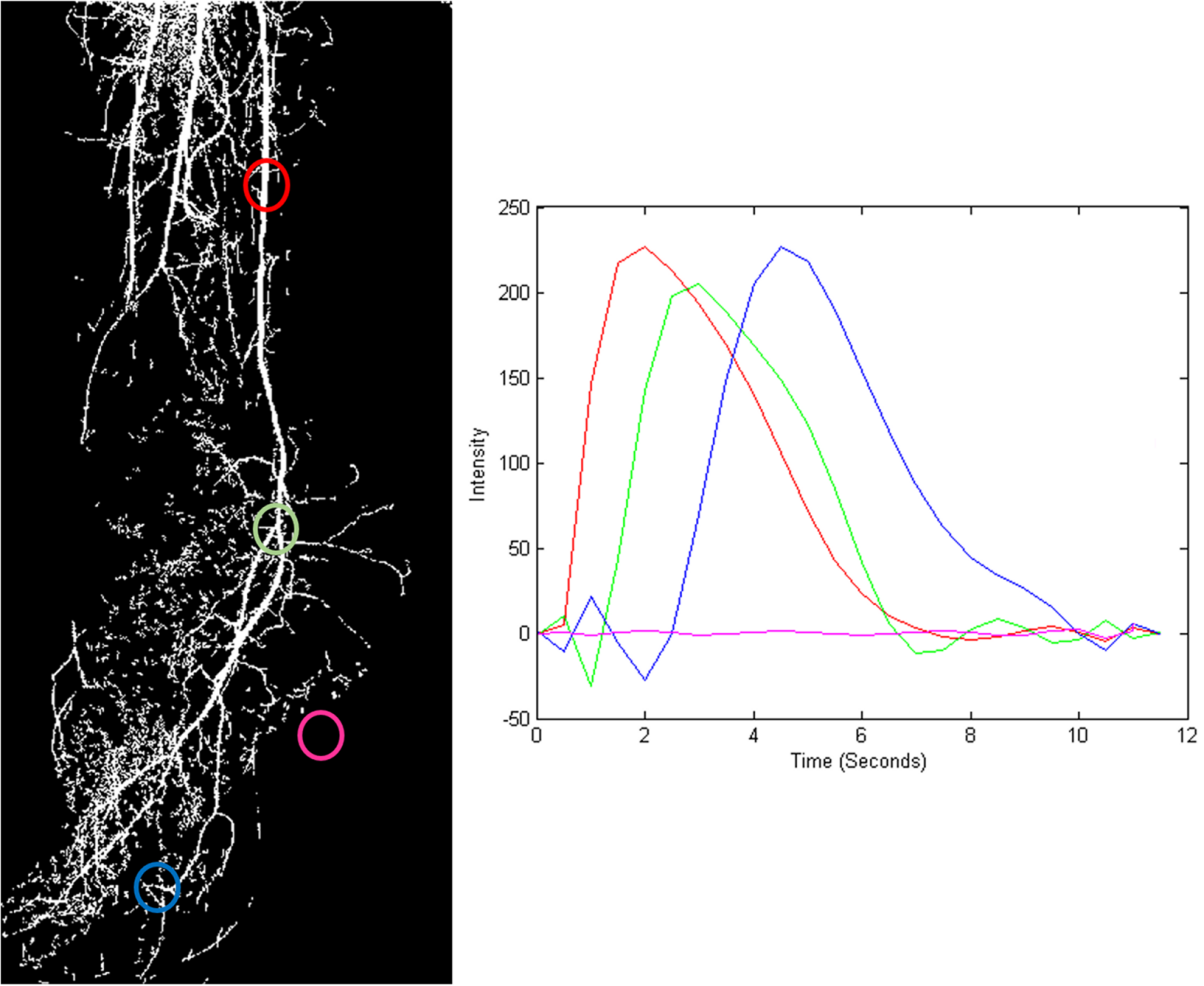
Time-intensity curve calculation in masked concentration slices from various positions shown in red, green, blue, and pink. The perfusion blood volume and mean transit time maps were then used for perfusion blood volume map extraction according to Eq. (2)

In order to calculate MTT map on the masked image (detected vessels), we applied the vessel mask on each slice of the complement image set. Then, on the resulting image set, the TICs for each pixel in position (*x*, *y*) across time (slices) were plotted along with their grade 5 polynomial approximation (Fig. 4). Subsequently, for each polynomial curve, the MTT was calculated as the “full width half maximum” of the curve, here specified as the Euclidian distance between the two points of the TIC where it gets the half of its maximum value.

2 $$ \mathrm{CBF}\kern0.5em =\kern0.5em \frac{\mathrm{CBV}}{\mathrm{MTT}} $$

**Fig. 5:**
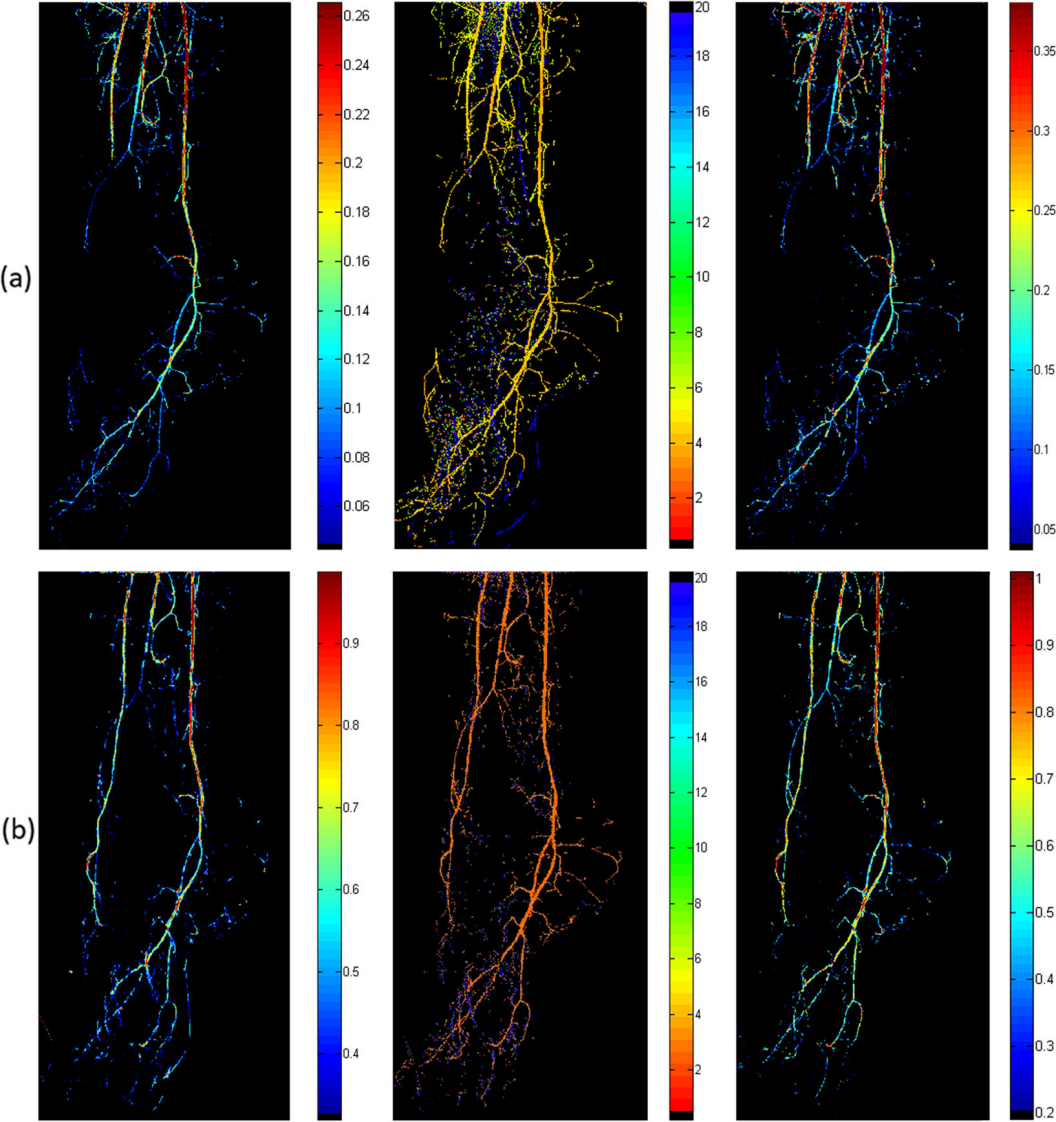
**a** Preoperative PBV, MTT, and PBF maps (from left to right). **b** Postoperative PBV, MTT, and PBF maps (from left to right). *PBV* Perfusion blood volume, *MTT* Mean transit time, *PBF* Perfusion blood flow

**Fig. 6:**
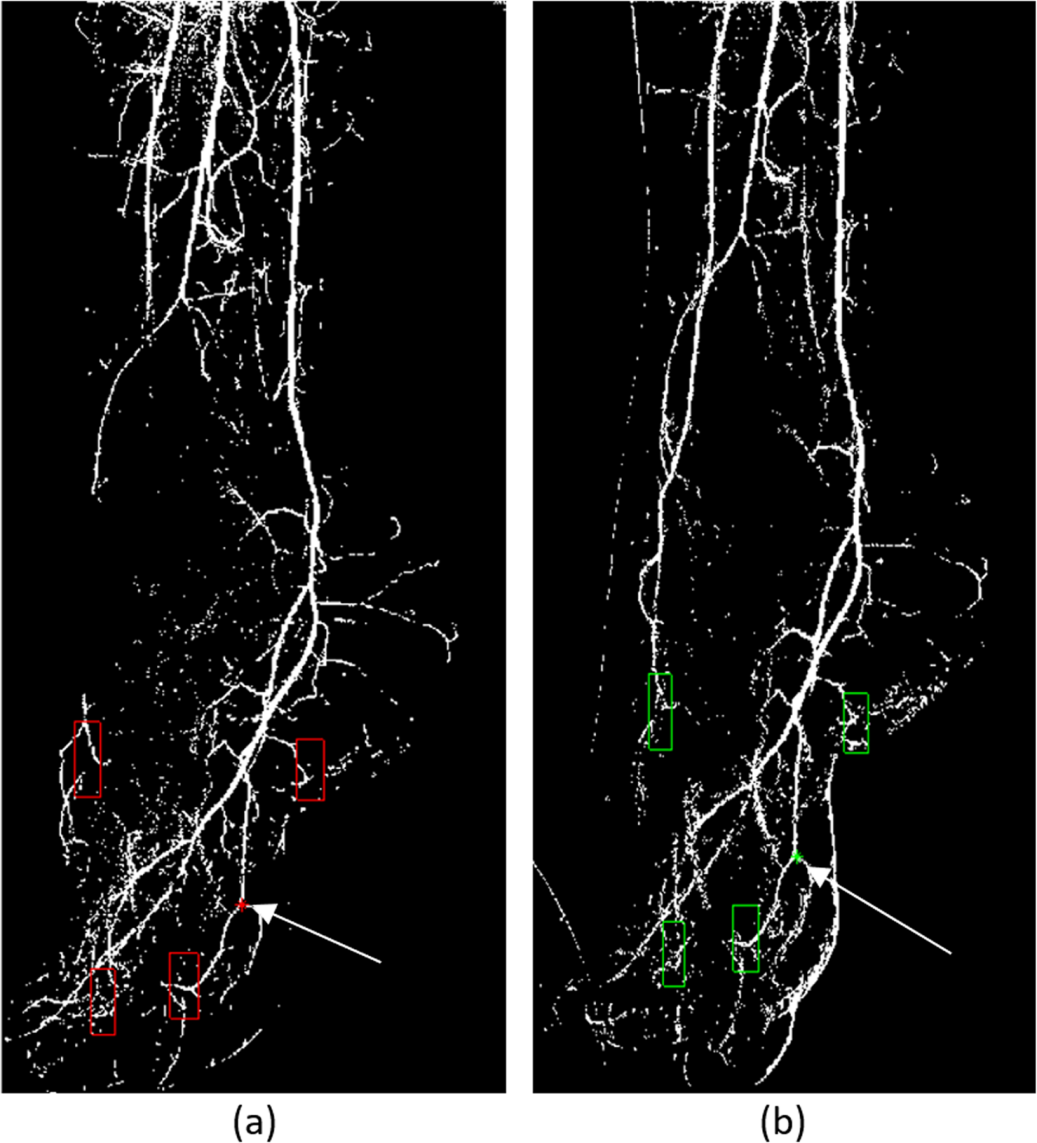
Region of interest (ROI) initialization for analysis and marked registration points. **a** Pre-therapy with ROIs and registration point in red (arrow) and post-therapy with ROIs located by the registration procedure and registration point in green (arrow)

For each patient, PBV, MTT, and PBF maps were acquired before and after treatment (Fig. 5). Several ROIs are then selected by the user in all perfusion maps to calculate their mean value towards patients’ treatment progress evaluation. However, as pre-therapy and post-therapy DSA image sequences are acquired by different examinations, the position and direction of patient’s foot may appear slightly different. A manual, point-based matching registration procedure was employed in order the ROIs defined for comparison to refer as much as possible to the same areas. The user defined the ROIs at the pre-therapy mask along with a point that clearly exists in both pre- and post-therapy masks. Then, the same point was marked on the post-therapy mask. The algorithm calculated the relative distance coordinates between the user-defined ROIs and the point defined in the pre-therapy mask. The positions of the corresponding ROIs at the post-therapy mask were then calculated employing the same relative distance coordinates (Fig. 6).

In order to assess the repeatability and reproducibility of the proposed method, additional measurements were performed by two radiologists with 15 and 20 years of experience in endovascular procedures. As regards the repeatability of the measurements, 10 times repetition of the algorithm was performed using the exact same ROIs (FCM initialization and pre-therapy and post-therapy clinically significant ROIs). As regards the intraobserver and interobserver variability, the already participating radiologist defined new ROIs on the same patients, while a new radiologist with 2 years of experience in endovascular procedures was asked to select new ROIs as well. In total, three interventional radiologists were involved in this study. For the two sets of pre-therapy and post-therapy measurements, the intraclass correlation coefficient (ICC) was calculated for each parameter (CBF, CBV, MTT).

## Results

Among ten consecutive patients, seven fulfilled the selection criteria and five patients were included in the study, as image post-processing was not feasible in two patients due to significant motion artifacts produced during DSA, despite attempts to achieve an optimal target limb immobilization giving a technical success of 5/7 (71.5%). All patients suffered from diabetes mellitus and CLI. Patients’ age was 54 ± 16 years (mean ± standard deviation), ranging from 28 to 72 years. Patients’ baseline demographics, procedural details, and outcomes are analytically reported in Table 1. Procedural success was 7/7 (100%).

In these five patients, pre- and post-revascularization 2D perfusion imaging; calculation of MTT, PBF, and PBV values; and quantification of the variation of post-procedural tissue perfusion were successfully performed (Table 2). Following ROI selection, mean image post-processing time was 7 ± 2 min (mean ± standard deviation), ranging from 4.4 to 9.3 min, using Matlab R2014a in a PC with an Intel(R) Core (TM) i7-5820K CPU @3.30 GHz, 16 MB RAM, and an nVidia GeForce 970 GTX GPU. Balloon angioplasty was successful in all cases. Post-procedural MTT was decreased in four out of five patients demonstrating an increase in tissue perfusion after revascularization.

**Table 2:** Preoperative (Io) and postoperative (I) values of MTT, PBF, and PBV along with the postoperative percent rise on the user-defined ROIs set on ATA and PA areas for each of the five patients

Patient 1	Before (*I*_0_)		After (*I*)	% rise 100 × (*I* − *I*_0_)/*I*_0_	Patient 2	Before (*I*_0_)		After (*I*)	% rise 100 × (*I* − *I*_0_)/*I*_0_
	Artery	ROI				Artery	ROI		
MTT	ATA	5.89	5.20	-10.81		ATA	17.25	5.77	-66.56
	PA	4.58	10.21	123.10		PA	15.76	7.27	-66.56
	PA	8.19	8.52	4.10		PA	16.01	10.85	-66.56
	PA	8.05	8.78	9.05		PA	19.00	11.79	-66.56
PBF	ATA	0.04	0.14	232.26		ATA	0.015	0.08	404.57
	PA	0.11	0.08	-26.39		PA	0.021	0.04	220.93
	PA	0.05	0.12	131.10		PA	0.024	0.08	233.61
	PA	0.05	0.09	79.47		PA	0.00	0.06	1,453.85
PBV	ATA	0.20	0.39	96.22		ATA	0.22	0.39	75.52
	PA	0.49	0.29	-40.58		PA	0.17	0.24	39.80
	PA	0.26	0.38	43.35		PA	0.36	0.41	14.07
	PA	0.24	0.29	19.11		PA	0.07	0.47	529.45
Patient 3	Before (*I*_0_)		After (*I*)	% rise 100 × (*I* − *I*_0_)/*I*_0_	Patient 4	Before (*I*_0_)		After (*I*)	% rise 100 × (*I* − *I*_0_)/*I*_0_
	Artery	ROI				Artery	ROI		
MTT	ATA	12.71	4.56	-64.16		ATA	12.43	8.83	-28.98
	PA	13.14	11.66	-11.23		PA	13.28	8.38	-36.92
	PA	15.75	10.29	-34.68		PA			
	PA					PA			
PBF	ATA	0.03	0.19	3,360.29		ATA	0.04	0.05	18.59
	PA	0.02	0.03	39.09		PA	0.04	0.03	-23.76
	PA	0.02	0.09	290.00		PA			
	PA					PA			
PBV	ATA	0.37	0.86	131.05		ATA	0.41	0.41	0.02
	PA	0.18	0.16	-11.02		PA	0.38	0.23	-41.28
	PA	0.27	0.53	95.39		PA			
	PA					PA			
Patient 5	Before (*I*_0_)		After (*I*)	% rise 100 × (*I* − *I*_0_)/*I*_0_					
	Artery	ROI							
MTT	ATA	13.09	5.35	-59.15					
	PA	14.56	5.57	-61.72					
	PA								
	PA								
PBF	ATA	0.04	0.09	140.53					
	PA	0.03	0.07	135.78					
	PA								
	PA								
PBV	ATA	0.28	0.45	59.53					
	PA	0.28	0.37	34.07					
	PA								
	PA								

*PBV* Perfusion blood volume, *MTT* Mean transit time, *PBF* Perfusion blood flow, *ATA* Anterior tibial artery, *PA* Posterior tibial artery

In patient 1, a 10.8% decrease in MTT was noted only in one out of the four selected areas of interest (ROI number #1) and was correlated with the foot area of the artery treated (Table 2). An increase of MTT was noted in ROI number #2 (-123.1%) and a less pronounced increase in ROI number #3 (4.1%) and number #4 (9.0%) (Table 2). In the remaining four patients, a decrease in MTT post-revascularization and therefore an increase in tissue perfusion were noted in all selected ROIs. PBF and PBV values were also analogously increased following revascularization in all five patients and in the vast majority of selected ROIs: 13/15 ROIs (86.6%) and 12/15 ROIs (80%), respectively. All MTT, PBF, and PBV values and the quantification of tissue perfusion post-revascularization in each patient are analytically reported in Table 2. No procedure-related complications were noted. Limb salvage and complete wound healing were observed for 4/5 limbs (80.0%) at 6 months of follow-up.

As regards the algorithm’s repeatability after the 10 times repetition, the exact same results were presented. The ICC was found to be 0.92 for CBF, 0.91 for CBV, and 0.87 for MTT for the intraobserver variability and 0.84, 0.86, and 0.82 for the interobserver variability, respectively.

## Discussion

The proposed custom-made, 2D perfusion DSA algorithm was safe and achieved the quantification of infrapopliteal angioplasty for the treatment of CLI, with a technical success rate of 72%.

A MTT decrease after angioplasty was noted in four out of five cases, indicating an increase of tissue perfusion following successful revascularization. This was reflected in the clinical outcomes, as wound healing was achieved in four out of five cases at 6-month follow-up. Moreover, pre-procedural and post-procedural variation of MTT was quantified, and the percentage of increase or decrease of foot perfusion following endovascular revascularization was calculated. Notably, an increase in tissue perfusion was noted in all ROIs directly supplied by the revascularized arteries. However, in patient #1, in which pedal arch occlusion revascularization was not technically feasible, an increased tissue perfusion was detected only in the area supplied by the revascularized anterior tibial artery (Fig. 6a, ROI #1). Moreover, in this case, the perfusion calculated in ROIs supplied by the posterior circulation at the level of the heel was decreased after angioplasty of the anterior tibial artery (Fig. 6a, ROIs #2, #3, and #4). The authors speculate that this phenomenon could be explained either by a technical error owned to a mismatch between pre-procedural and post-procedural ROIs attributed to unperceivable limb movement or by the fact that angiosome-targeted revascularization of the occluded anterior tibial artery and the absence of communication between the anterior and posterior circulation led to redistribution of foot perfusion favoring the recently revascularized anterior tibial artery area. On the other hand, in patient #2, in involving both the anterior and the posterior circulation (Fig. 6b). This is in line with previous studies indicating the quality of the pedal arch significantly influences wound healing rather than angiosome-directed angioplasty [16].

Finally, in patient #5, no arch was visible after revascularization and an increase in perfusion was noted only in restricted tissue areas around the revascularized anterior tibial artery. This patient underwent major above the knee amputation 1 month after the procedure, further highlighting the fact that the achievement of vessel patency is not synonymous to clinical success, as well as the impact of pedal arch outflow for limb salvage, as previously reported [17].

In this study, CBF, CBV, and MTT parameters were extracted from TIC curves and evaluated for patients’ condition improvement having CLI. These parameters were computed by means of a custom-made FCM-based segmentation algorithm that manages to detect only vessel information towards performance improvement and processing time decrement. The proposed segmentation algorithm omits non-valid information from vessel background and could be independently used to each commercial software.

As regards available software packets, differently from our study, most approaches employ parameters from TICs such as “area under the curve” and “peak concentration.” The aforementioned computed parameters were also employed by Galanakis et al. [18] in a relatively small sample of patients. Both results from the two studies are in agreement, demonstrating clinical importance and feasibility.

In addition, the properties of the proposed segmentation algorithm provide the employment of small ROIs on the vessel mask for analysis differently from other software packets that process information regarding lower foot area derived from large ROIs [13, 14, 18–20]. Other studies in the literature also tried to quantify DSA perfusion angiography on patients with diabetes using quantification parameters extracted by TICs using appropriate software implementations [13, 14, 19, 20]. These works have shown that quantification techniques using the aid of specialized software can monitor successfully patients’ condition and provide a useful tool for clinicians. Although these studies’ approach is similar to that here proposed, a direct comparison cannot be made due to the small sample enrolment, absence of a “reference standard” for segmentation, and different quantification parameters.

The proposed method is a semiautomatic reader-dependent method which needs some experience of use. As regards the registration procedure, it is mandatory only when substantial differences between DSA examinations are present. To avoid this processing time-expensive additional step, specific foot casts are under construction to standardize and ensure reproducibility of the pre-DSA and post-DSA imaging procedure. When these differences between pre-DSA and post-DSA image sets are omitted, the algorithm can be used in a real-time analysis. Regarding the current dataset and the radiologist’s experience, a small amount of training was necessary since the majority of ROIs’ selection was successful without the need of repetition.

This feasibility study has limitations. The most important one is that the number of patients investigated was small. Therefore, regardless of the aforementioned promising results, the small number of limbs included in the analysis deteriorates the generalization properties of the proposed approach. Another limitation is that visual estimation and not quantitative vessel analysis was used in order to assess procedural success. Nevertheless, procedural success was a secondary endpoint of this study, while both visual estimation and quantitative vessel analysis can only assess patency of the treated vessel and not the hemodynamic parameters of foot perfusion that were quantified by the proposed angiography perfusion software. As regards the vessel mask extraction procedure, it is dependent on the user-specified ROIs leading to initialization values fed to the FCM algorithm. The FCM variant used in this study is a pixel-based clustering method that suffers from noise and intensity variations that may categorize pixel areas or objects to the same cluster when the desired outcome is different. For some problems such as the construction of the vessel mask in this study, a preinitialization of the clusters’ centers. is required. ROIs taken on fully perfused vessels with high intensity values may lead to under-segmentation of small vessels that are not fully perfused and therefore reach lower intensity values. As there was no reference standard used regarding vessel extraction, there is no indication regarding the performance of the segmentation algorithm process. Regarding the TIC extraction and MTT calculation process, it is dependent on the total frame number and frames per second rate. Although a polynomial fit is applied to the TICs, small differences between estimated and real values should be expected. The registration process is also dependent on user-defined points that indicate the same anatomical areas on pre-therapy and post-therapy vessel masks. Since the mean intensity values of PBV, MTT, and PBF of the chosen ROIs were calculated, relatively large enough ROIs should be selected to minimize calculation errors due to unmatched areas. It should be mentioned though that the vessel extraction process is implemented for lesser computational times and ease of pre-therapy and post-therapy image evaluation process, assuming that the aforementioned limitations do not affect diagnosis. Limb immobilization was essential for image acquisition and post-processing, as movement artifacts do not allow correct post-processing of the acquired images. For this reason, perfusion imaging was not feasible in two patients as adequate immobilization using a soft strap could not be achieved. Perhaps the use of more efficient immobilization methods could expand the applicability of the method even in less cooperative patients, while this method would be easy to perform in cases that have been selected to undergo revascularization under general anesthesia.

Due to the relevant technological improvement of noninvasive imaging modalities, currently recommended pre-procedural and post-procedural imaging for patients undergoing peripheral endovascular interventions includes duplex ultrasound, computed tomography angiography, and magnetic resonance angiography. Intra-arterial DSA is reserved only for intra-procedural imaging and in cases of unresolved radiological issues with noninvasive imaging. However, the proposed algorithm could be used in general clinical applications and other noninvasive modalities such as magnetic resonance techniques, where a similar protocol as that used in this study could be utilized by the proposed algorithm for quantification purposes.

The proposed algorithm produced the exact same results after the 10 times repetition. This is attributed to the vessel and background ROIs’ selection for FCM initialization that leads to the same local maxima detection and finally the calculation of the same cluster centers. As regards the intraobserver and interobserver variability, the ICC calculation showed excellent agreement for the intraobserver variability and good agreement for the interobserver variability for each parameter (CBF, CBV, and MTT). This shows that the proposed method may be used as an objective alternative to visual inspection.

In conclusion, quantification of infrapopliteal angioplasty outcomes using this newly proposed, custom-made, intra-procedural PDSA algorithm was performed using PBV, MTT, and PBF maps. Limb immobilization remains a challenge especially in non-cooperative patients. Further studies are deemed necessary to determine its clinical role in peripheral endovascular procedures.

## Data Availability

The datasets used and/or analyzed during the current study are available from the corresponding author on reasonable request.

## Abbreviations

2D: Two-dimensional
3D: Three-dimensional
CLI: Critical limb ischemia
DSA: Digital subtraction angiography
FCM: Fuzzy C-means
MTT: Mean transit time
PAD: Peripheral arterial disease
PBF: Perfusion blood flow
PBV: Perfusion blood volume
ROI: Region of interest
TIC: Time-intensity curve
